# Intensification of tidally generated internal waves in the north-central Bay of Bengal

**DOI:** 10.1038/s41598-020-62679-4

**Published:** 2020-04-08

**Authors:** A. K. Jithin, M. P. Subeesh, P. A. Francis, S. S. V. S. Ramakrishna

**Affiliations:** 1 Indian National Centre for Ocean Information Services (INCOIS), Hyderabad, 500090 Telangana India; 2 National Centre for Polar and Ocean Research (NCPOR), Vasco-da-Gama, Goa 403 804 India; 3 Department of Meteorology and Oceanography, Andhra University, Visakhapatnam, 530003 Andhra Pradesh India

**Keywords:** Ocean sciences, Physical oceanography

## Abstract

Flow of barotropic tidal currents over topographic features, such as continental slopes and submarine ridges, generates internal gravity waves at tidal periods known as internal tides. Amplitude of these waves are generally large near the generation regions. Analysis of Sea Surface Height (SSH) data, derived from satellite altimeter revealed the amplification of internal tides in the semidiurnal period in the north-central Bay of Bengal (BoB) (around 89${}^{\circ }$E, 16${}^{\circ }$N), which is about 450 km away from their generation sites. SSH signals found in the north-central BoB ($\sim$3 cm) were comparable to the maximum amplitudes (2.5 to 3.5 cm) observed near their potential generation sites in the BoB such as continental slopes in the head of the bay and Andaman-Nicobar (AN) Ridge. Simulations from a high-resolution regional ocean model also confirmed the presence of large internal tide amplitude in the north-central BoB. Our study revealed that convergence of internal tides, which were generated along the concave-shaped source (continental slopes in the head of the bay and the northern parts of AN Ridge), into its focal region caused their amplification in the north-central BoB. It was also found that internal tide energy dissipation rates in this focal region were about 10 times larger than those in other open ocean regions.

## Introduction

Periodic flow of tidal currents over steep topography generates vertical oscillations of isopycnal surfaces in the stratified ocean. These internal oscillations propagate away as gravity waves, known as internal tides. Continental margins and submarine ridges are the main sources of this tidally-generated internal gravity waves in the ocean. Previous studies suggest that about 30% of the energy associated with internal tides, especially with high vertical modes, dissipates locally near the generation sites themselves and the remaining 70% of the energy radiates from the source, propagates long distances (O (1000 km)) and ultimately dissipates in deep ocean or in remote continental margins^1–3^. Dissipation of internal tides is one of the important sources of mechanical energy for the vertical mixing in the interior ocean^4^. Several mechanisms have been suggested for the dissipation of long-range propagating internal tides such as interactions with rough topography^5^, interactions with mean flows and eddies^6^, cascade to smaller scales via wave-wave interactions^7^ etc. However, the pathways of internal tide energy in the ocean and regions of their energy dissipation are not completely understood.

Generally, strong baroclinic currents and large isopycnal displacements at tidal frequencies are observed near their generation sites and their amplitude decreases away from the sources^8,9^. However, spatial variation of internal tides and associated energy dissipation in the ocean can be more complicated due to the interaction of waves from multiple sources^10^. For example, Wang *et al*.^11^ reported that interference of internal tides from different sources results in large spatial inhomogeneity in the energy flux and its dissipation in the Philippine Sea. Therefore, understanding the spatial distribution of internal tides is very important to interpret their characteristics and associated mixing using the *in-situ* measurements and satellite altimeter data.

**Figure 1:**
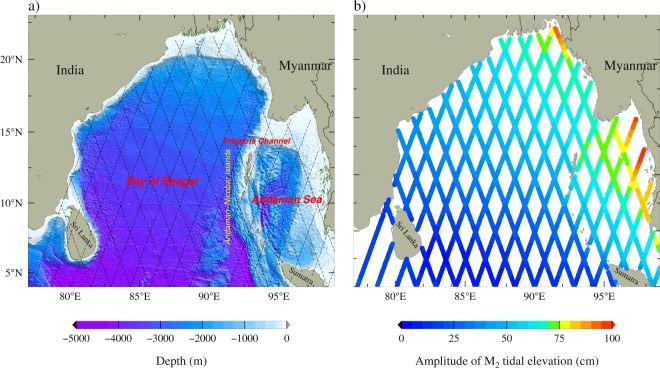
(**a**) Map shows the model domain and bathymetry of the Bay of Bengal. Black dashed line represents the ground tracks of TOPEX/Poseidon (T/P), Jason-1 (J1) and Jason-2 (J2) satellites. Bathymetric contours (brown) of 100 and 1000 m are also shown. (**b**) Amplitude of M${}_{2}$ tidal elevation along the satellite tracks.

Bay of Bengal (BoB, Fig. 1) is a semi-enclosed tropical bay located in the north-eastern flanks of the Indian Ocean, which is one of the geographical regions where the characteristics of internal tides are least studied. Most of the internal tide activity in the BoB is confined to semidiurnal frequencies such as M${}_{2}$ and S${}_{2}$ (principal lunar and solar semidiurnal tidal constituents respectively). This is due to the dominant semidiurnal nature of barotropic tides in this region, that primarily generate internal tides^12^. Andaman-Nicobar (AN) Ridge is the most important source of semidiurnal internal tides in this region with a depth-integrated semidiurnal M${}_{2}$ energy flux upto 30 kW m^−1^^13,14^. Recent observational programmes such as Ocean Monsoon Mixing-Air-Sea Interactions Regional Initiative (OMM-ASIRI) to study the oceanic processes in the BoB, including turbulence measurements suggest that internal waves may play an important role in the vertical mixing across the density layers (diapycnal mixing) in the deep BoB^15,16^. Therefore, for interpreting the mixing processes in the deep ocean, it is very important to take into account the spatial variability of internal tides and their energy pathways^10^, which is poorly understood in the BoB.

One of the main difficulties in studying the internal tides is the limited spatial coverage of *in-situ* observations. However, Ray and Mitchum^17^ demonstrated that satellite altimeters could detect Sea Surface Height (SSH) signals of the order of a few centimeters corresponding to subsurface oscillations of isopycnals due to internal tides. In this study, we use SSH data along the satellite tracks (Fig. 1a) derived from the combined altimetry records of TOPEX/Poseidon (T/P) satellite, Jason-1 (J1) and Jason-2 (J2) to examine the spatial variability of internal tides in this region. One of the interesting features we noticed is the presence of large signals of internal tides in the north-central BoB, which is hundreds of kilometers away from their generation sites. This motivated us to investigate the mechanisms of this observed intensification of internal tides with the help of simulations from a numerical model based on Regional Ocean Modelling System (ROMS). We also examined the implications of these high amplitude internal tides in this region.

## Results

### Observed internal tide signals from satellite altimeter data

**Figure 2:**
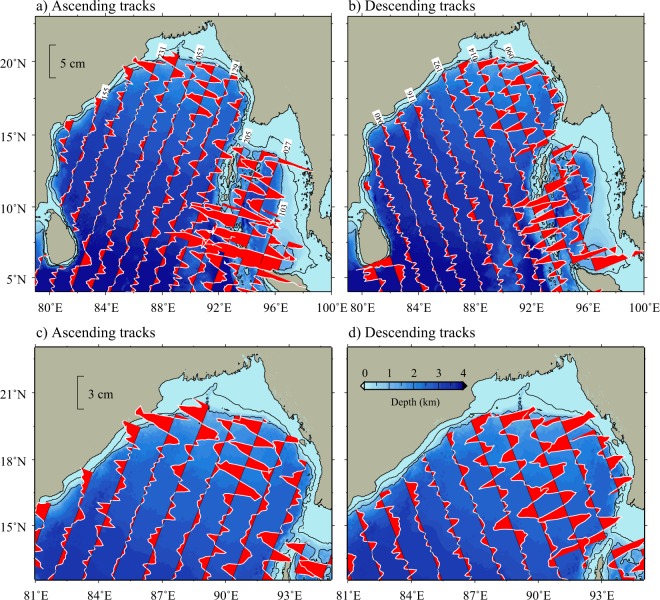
SSH signals associated with M${}_{2}$ internal tides in the Bay of Bengal derived from satellite altimeter data along (**a**) ascending and (**b**) descending tracks respectively. Bottom panels (c and d) are the zoomed view of SSH signals in ascending and descending tracks in the northern BoB. Bathymetric contours of 100 and 1000 m is also shown.

Semidiurnal M${}_{2}$ is the largest tidal constituent in the BoB (Fig. 1b) followed by S${}_{2}$ and their spatial variation is almost similar^18^. Therefore, we restricted the analysis only to M${}_{2}$ internal tides in this paper. Figure 2 shows surface expressions of M${}_{2}$ internal tides in the BoB derived from satellite altimeter data. SSH signals in the shallow regions (with depth less than 500 m) were removed from the analysis due to relatively low accuracy of altimeter data near the coastal regions^19^. It may be seen from the figure that internal tides are present all over the BoB with relatively large SSH signals with amplitudes of about 2.5 to 3.5 cm near the AN Ridge and adjacent to the continental slopes in the northern BoB (Fig. 2). Note that surface signals with similar amplitudes are also observed in the Andaman Sea (2.5–4 cm). The altimeter observations confirm the presence of energetic coherent internal tides near the AN Ridge and the northern BoB which are the main internal tide generation sites in this region^14^. The observed M${}_{2}$ amplitudes near the northern BoB and the AN Ridge are comparable with those observed near the strong internal tide generation sites in the world ocean, such as, Hawaiian Ridge^20^ (~3 cm) and Luzon Strait^21^ (~5 cm). Further, wavelengths of this along-track SSH signals are about 100–150 km in the BoB (Fig. 2). Small amplitudes of SSH signals in the western and central BoB indicate weak internal tide activity in these regions.

Large amplitudes of internal tide signals are often expected near the topographic features such as continental slopes and ridges. However, a striking feature to note in the distribution of SSH signals is the presence of large amplitudes (2–3 cm) of M${}_{2}$ internal tides in the deep regions of the north-central BoB (around 16${}^{\circ }$N and 90${}^{\circ }$E in Fig. 2). It is interesting to note that the amplitude of M${}_{2}$ component of internal tides in this region is even larger than those near the continental slope of the northern BoB in some of the descending satellite tracks (for example, track 014 in Fig. 2c). This suggests that internal tides get amplified in the north-central BoB. We use simulations from a high-resolution model (ROMS) to understand the generation and propagation of internal tides and to identify the mechanism for their observed amplification in the north-central BoB (see data methods for more details).

### Surface signatures of internal tides from the model simulations

**Figure 3:**
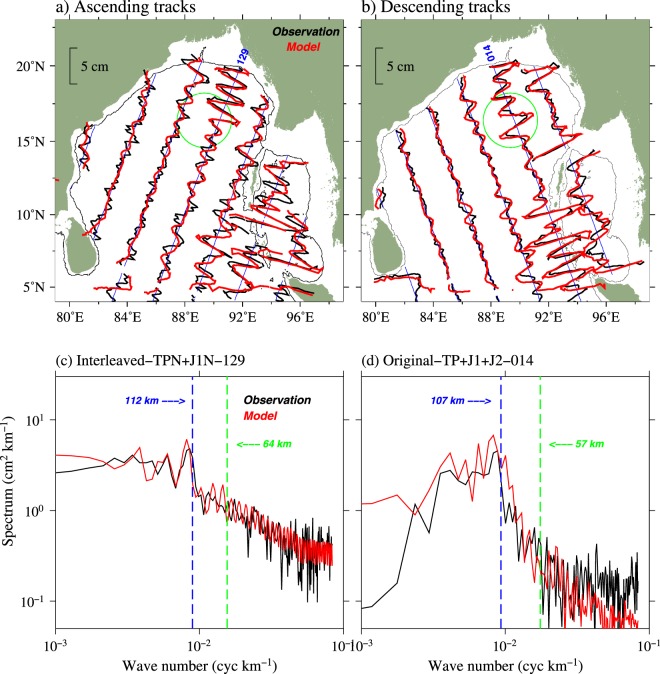
Comparison of SSH signals associated with M${}_{2}$ internal tides in the BoB derived from observation (black) and model (red) along (**a**) ascending and (**b**) descending satellite tracks. Only alternative tracks are shown to avoid cluttering. Bathymetric contour of 1000 m is also shown. Green circle indicates the region where large surface signals of M${}_{2}$ internal tides in the north-central BoB. Wavenumber spectra of M${}_{2}$ SSH signals from the observation (black) model (red) along the satellite track (**c**) TPN − J1N − 129 (interleaved) and (**d**) TP + J1 + J2 − 014 are shown. Vertical dashed lines represent the average theoretical wavelength of the first (blue) and second (green) baroclinic mode for M${}_{2}$ internal tides along the satellite track computed by normal mode decomposition.

Amplitude of M${}_{2}$ surface signals from the model simulations agree well with the satellite observations (Fig. 3). The spatial variability of M${}_{2}$ internal tides in the BoB, including large surface amplitude for M${}_{2}$ in the deep regions of the northern BoB centered around 90${}^{\circ }$E, 16${}^{\circ }$N were simulated very well by the model. Further, filtered SSH signals from altimeters and model simulations along the selected satellite tracks (TPN + J1N − 129 and TP + J1 + J2 − 014) were subjected to spectral analysis to obtain the wavelength of these signals. The wavenumber spectra of M${}_{2}$ signals from the altimeter and model data show a prominent peak at a wavelength of about 120 km (Fig. 3c, d), which is consistent with the theoretical wavelength of the first baroclinic mode of M${}_{2}$ internal tide in the BoB. In addition, M${}_{2}$ wavenumber spectra have only a single peak around the first mode wavelength (~112 km) and there are no significant peaks corresponding to the second or other higher modes along these tracks. Empirical Orthogonal Function (EOF) analysis on the model simulated semidiurnal baroclinic velocities at locations near (87.7${}^{\circ }$E, 19.25${}^{\circ }$N) and far from the generation sites (85.79${}^{\circ }$E, 17.49${}^{\circ }$N) in the head of the bay was carried out to quantify the relative contributions of each of the vertical modes. This analysis shows that about 88% of the total variance of semidiurnal baroclinic velocity is contributed by the first EOF mode in both the near and far field regions. The vertical structure of first EOF mode of velocity fields near and far-field locations (figure not shown) resembles to the first baroclinic mode of internal tides. This suggests that M${}_{2}$ internal tides in the BoB are dominated by the first baroclinic mode, and higher modes (mode 2 and higher) are relatively weak (12%). It may be noted that the wavelengths of internal tides slightly vary among the satellite tracks (Fig. 3). Both simulated and observed wavenumber spectra show that the spectral peaks are relatively wide in track 014 (Fig. 3d), which could be associated with the direction of propagation of internal tides from AN Ridge (which is oblique to the satellite track) as indicated in Zhao *et al*.^22^.

### Intensification of internal tides in the north-central BoB: the mechanism

**Figure 4:**
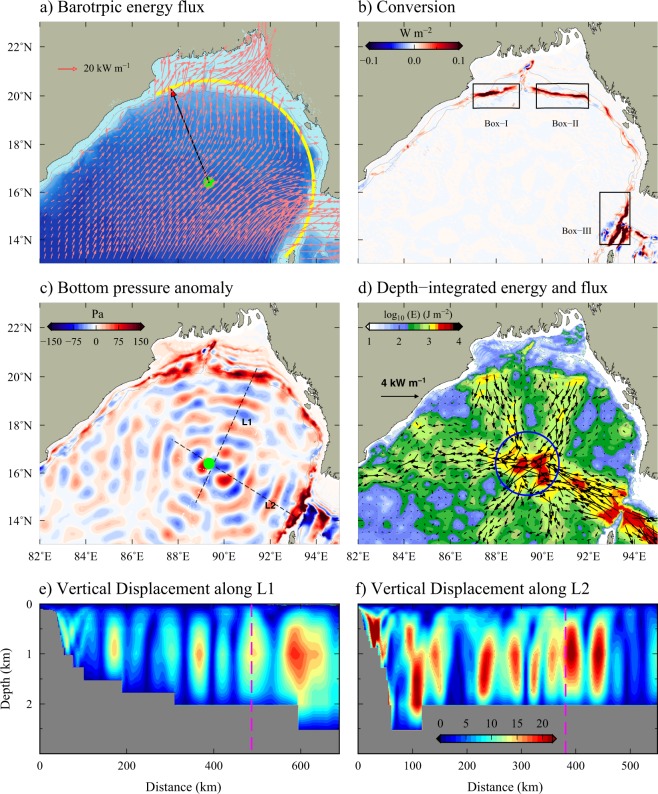
Barotropic M${}_{2}$ flux vectors in the northern BoB. Yellow curve indicate the arc-shape of the continental slope in the northern BoB and black arrow shows the radius (457 km) of the arc. (**b**) Barotropic to baroclinic M${}_{2}$ conversion rates. Boxes (I–III) represent the major internal tide generation sites. (**c**) Baroclinic M${}_{2}$ bottom pressure anomaly (Pa) at the bottom and (**d**) total energy (E = APE + HKE) of M${}_{2}$ internal tides. Blue circle in (**d**) represent the focal region. Bathymetric contours of 100 and 1000 m are shown. (**e**,**f**) Vertical displacement of isopycnals associated with M${}_{2}$ internal tides along the transect L1 (**e**) and L2 (**f**) shown in (**c**). All the computations are performed using one-month (September 2013) hourly model output.

Generally, large internal tide signals in the open ocean occur primarily due to the local generation over submerged ridges or seamounts. Bottom topography in the north-central BoB is relatively smooth and no seamounts or ridges are present in this region. Therefore, the possibility of local internal tide generation by underwater topography can be ruled out (Fig. 4a). This suggests that internal tides observed in the north-central BoB come from distant sources. In order to identify the generation sites of internal tides in the northern BoB, we estimated barotropic to baroclinic energy conversion rates from the model simulations (Fig. 4b). Positive conversion rate indicates the transfer of energy from barotropic tides to internal tides. Spatial map of conversion rates shows that continental slopes in the northern BoB and shallow passage north of AN Ridge (Preparis channel) are the nearby source of internal tides, where large amount of barotropic energy gets converted to baroclinic (Fig. 4b). In the head of the bay, maximum energy conversion occurs on both sides of the submarine canyon (Box-I and II in Fig. 4b). Barotropic M${}_{2}$ flux vectors along the continental slope in the head of the bay and Preparis Channel are perpendicular to isobaths, which favours the efficient transfer of barotropic energy into internal tides (Fig. 4a).

These nearest sources of internal tide generation located along the continental slopes in the head of the bay and northern parts of the AN Ridge are more than 450 km away from the regions where large SSH signals are observed. It may be noted that, the shape of the continental slopes in this region can be nearly described by a semi-circle (Fig. 4a, yellow curve) with a radius (black arrow) of about 457 km centered at 89.35${}^{\circ }$E, 16.4${}^{\circ }$N (green dot in Fig. 4a). Internal tides radiate from the sources oriented in circular shape can be converged into their focal region^23^. Depth-integrated baroclinic energy and flux estimated from the model simulations clearly show the radiation of energy from the main generation sites and their convergence into the focal region located in the north-central BoB. This convergence results in the enhancement of internal tides in the north-central BoB. Baroclinic M${}_{2}$ energy fluxes in the regions surrounding to this focal point are as high as about 6 kW m^−1^ and energy is about 4 kJ m^−2^. Nearly uniform phases of the M${}_{2}$ baroclinic bottom pressure suggest that internal tides get generated at different sources along the slopes in northern BoB without significant delay in time (Fig. 4c). This is mainly due to the nearly same phase of the barotropic M${}_{2}$ tide in the northern BoB^18^. For example, the phase difference in the barotropic M${}_{2}$ tide at Box-1 and Box-III shown in Fig. 4b is less than 40 minutes. Further, time evolution of bottom pressure anomalies (see the animation in the Supplementary 1) clearly illustrate the radiation of internal tides into the focal region in the north-central BoB. Vertical isopycnal displacement associated with M${}_{2}$ internal tide in the focal region is about 22 m, which is equal to or even larger than the displacement seen near the generation sites (Fig. 4e, f). These vertical displacement of isopycnals of the order of a few tens of meters is consistent with the amplitude of SSH (2–3 cm) signal observed from the satellite altimeter.

It is important to note that similar patterns of focusing of internal tides due to the arc-shaped sources were reported in a few other places also. For example, internal tides generated along the Mariana Arc in the Pacific Ocean^23^, Vitoria-Trindade Ridge in South Atlantic Ocean^24^ cause similar focusing of energy. The intensification of internal tide energy in the north-central BoB does not occur at a single point, but it is distributed over a region. As described by Zhao and  D’Asaro^23^, radius of the focal region ($r$) can be estimated by, $r=1.22f\lambda$/$D$, where $f$ is the focal length the $D$ is the diameter of the aperture (here the extent of arc), $\lambda$ is the wavelength^25^. For the northern BoB, $f$ = 457 km and $D$ = 1250 km and $\lambda$ = 112 km (for first mode M${}_{2}$ internal tide). The estimated radius of the focal region is about 160 km and the strong M${}_{2}$ internal tidal energy found in the north-central BoB lies within this focal region (blue circle in Fig. 4d). Unlike observed in other parts of the world ocean, where the energy is distributed in the entire focal region from perfectly arc-shaped sources, for instance Mariana Arc^23^, internal tide energy in the north-central BoB are found to be distributed unevenly in the focal region. It is important to note that the strength of internal tides coming from the arc-shaped sources is not equal and these sources are not oriented in a perfect arc shape also. Therefore slight differences in the orientation of these sources as well as the differences in the magnitude of internal tides comes from these sources could be the reason for the uneven distribution of internal tide energy found in the focal region.

**Figure 5:**
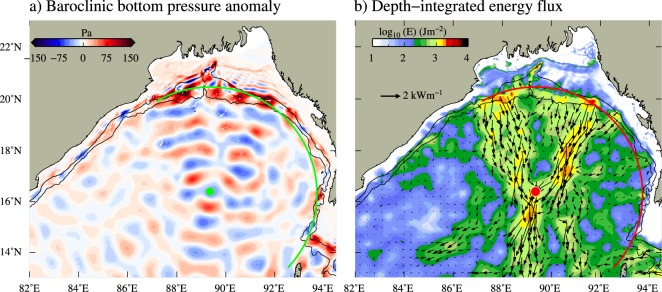
(**a**) Bottom baroclinic pressure anomaly and (**b**) depth-integrated energy overlaid with energy flux vectors of M${}_{2}$ internal tide from the experiment carried out by removing the AN Ridge from the model bathymetry. Green and red curves represent the imaginary arc with a radius of 457 km. Bathymetric contours of 100 and 1000 m are also shown.

Estimated energy conversion and flux show that Preparis Channel is the strongest source of internal tides compared to the other two sources in the head of the bay (Fig. 4). Therefore, the presence of AN Ridge and its orientation could have a profound impact on the distribution of internal tide energy in the northern BoB. To understand the contribution of internal tides originating from the Preparis Channel on their observed amplification in the north-central BoB, we carried out an experimental simulation by removing the AN Ridge from the model bathymetry. Note that since the AN Ridge and Islands are replaced with water, the amplitude of barotropic tides and hence the generation of internal tides in the head of the bay is slightly stronger in the experimental simulation compared to the realistic run. However, the spatial distribution of barotropic tides and internal tides are consistent in both the runs. Figure 5 shows the bottom pressure anomaly and depth-integrated energy flux of M${}_{2}$ internal tides from this experiment. As expected, the generation and westward propagation of internal tides from the AN Ridge were absent in the experimental simulation. However, southward radiating internal tides from the sources along the head of the bay were still directed into the focal region, which resulted in the enhancement of internal tide energy (Fig. 5). In the absence of AN Ridge, internal tides originating from the head of the bay focused into a more specific region in the north-central BoB. These southward radiating internal tides are found to be diverging beyond the focal region. This spatial pattern of convergence and divergence of internal tides and their amplification at the focal region is similar to the patterns observed near the Marina Arc^23^ and elsewhere.

Since the generation of internal tides greatly depends on the stratification of the ocean, energetics of internal tides can vary with season. We next examine how the variation in the intensity of internal tides associated with the changes in the stratification modulate their amplification in the north-central BoB. Northern BoB receives a large amount of fresh water discharge from adjacent continental rivers, which results in large near-surface stratification in this region, particularly towards the end of southwest monsoon (September-November)^26,27^. It may be noted that our analyses in the previous sections were based on the simulations for the month of September. Therefore, we estimated M${}_{2}$ internal tide energy and flux in the month of April when the stratification is relatively weak in this region (Supplementary 2). Internal tide energy fluxes originating from the head of the bay are found to be relatively weak (about 1.5 kW m^−1^) during April compared to September (See the Supplementary 2). This variation is mainly linked to changes in the internal tide generation which is due to the variation in background stratification in this region. It is found that, despite having differences in the strength of the internal tide beams originating from the major sources in April and September, the features such as focusing of internal tides and subsequent increase in their energy in the north-central BoB exist in both the months.

Further, the analyses carried out to delineate the role of internal tide-current interaction in this region show that the seasonal variability of background currents do not play a significant role in modulating the convergence of internal tides and associated intensification in the north-central BoB (Supplementary 2). However, the path of the internal tide beam beyond the focal region, especially which reaches continental margins of the western BoB, is found to be altered due to the presence of mesoscale eddies. This suggests that the path alteration of internal tide beam originating from the AN Ridge due to mesoscale fields could have a greater role in controlling the internal tide activity along the continental shelf and slope of the western BoB compared to north-central BoB. Similar path alteration of internal tide beam and associated change in internal tide energy due to the presence of mesoscale eddies was reported earlier in the South China Sea^28^.

### Effect of internal tide focusing on the distribution of energy dissipation in the deep ocean

**Figure 6:**
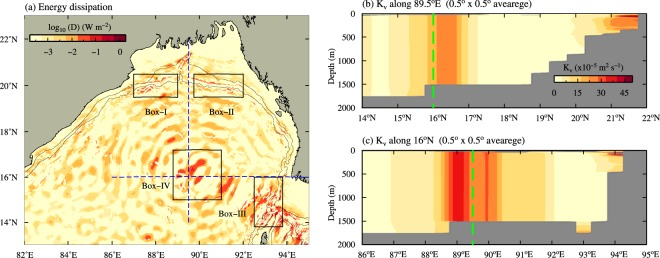
Depth-integrated dissipation rate of M${}_{2}$ internal tides in the northern BoB. Rectangular boxes represents the generation sites (Box-I–III) and focal region (Box-IV). Bathymetric contours of 100 and 1000 m are also shown. Blue dotted line shows the sections along the 89.5${}^{\circ }$E and 16${}^{\circ }$N. Eddy diffusivity (${K}_{v}$) computed using the internal tide parameterization (Eq. (1)) along (**b**) 89.5${}^{\circ }$E and (**c**) 16${}^{\circ }$N. Green dashed lines represent latitude/longitude in the north-central BoB, where the internal tide focusing occurs.

  Figure 6a shows the distribution of M${}_{2}$ internal tide energy dissipation rate ($D$) in the northern BoB estimated using the energy budget equation (see the data and methods for details). About 1363 MW of barotropic M${}_{2}$ energy is converted into baroclinic energy along the continental margins (50–1500 m) in the northern BoB, out of which about 372 MW (about 27%) dissipates near the generation sites themselves. Model estimates suggest that about 999 MW (72.6%) of baroclinic energy in the northern BoB radiates away and dissipates in remote areas. Some part of energy that radiates from the Preparis Channel even reaches continental margins off the east coast of India (Fig. 4). Relatively large dissipation rates are evident near the generation sites (Fig. 6a). Studies have shown that energy dissipation near the generation sites are mainly associated with the dissipation of internal tides having higher order vertical modes, while internal tides having lower order vertical modes radiate away and dissipate in remote regions^1^. However, it is important to note that higher M${}_{2}$ dissipation rates can be seen in the open ocean regions in the north-central BoB where the focusing of internal tides occurs (Box-IV in Fig. 6a). In this region, total energy dissipation is about 118 MW. The dissipation rates found in the focal region are about 10 times larger than the dissipation rates at other deep regions of the BoB and comparable to those in the generation sites in the northern BoB. This suggests that apart from the generation sites, enhanced internal tide dissipation can also occur in the open ocean due to energy convergence.

St. Laurent *et al*.^1^ proposed a parameterization scheme to estimate eddy diffusivity (${K}_{v}$) associated with internal tide dissipation (hereafter LSJ02). This scheme was originally designed to parameterize the enhanced turbulent mixing due to internal tides near the generation sites. Here we adopt a modified version of LSJ02, which is used to parameterize the internal tidal mixing in the Indonesian Seas^29^ and the Yellow Sea^30^, given by

1 $$\begin{array}{rcl}{K}_{v} & = & \Gamma \frac{qE(x,y)}{\rho \int {N}^{2}dz}+{K}_{0},\ when\ dN/dz < 0\\  & = & \Gamma \frac{E(x,y)}{\rho N}+{K}_{0},\ when\ dN/dz > 0\end{array}$$

where $\Gamma$ is the mixing efficiency, considered as 0.2, $q$ is the local dissipation efficiency (0.3) and E(x, y) is the barotropic to baroclinic energy conversion. Here, $N$ is the buoyancy frequency, $\rho$ is the density, $x$ and $y$ is the longitude and latitude coordinates. Background diffusivity (${K}_{0}$) is assumed to be a constant 10${}^{-5}$ m${}^{2}$s${}^{-1}$. In LSJ02 scheme, $qE(x,y)$ represents a fraction (about 30%) of energy (which is converted from barotropic to baroclinic) dissipated in the generation sites. The rest of the energy radiate away, eventually dissipate in remote regions, which were not considered in this parameterization scheme. But, here we use dissipation rate ($D$) obtained from energy budget calculation (see the data and methods) instead of $qE(x,y)$ in Eq. (1) as suggested by Liu *et al*.^30^, which accounts for the dissipation near the generation sites as well as remote regions. Figure 6b and c show the vertical structure of eddy diffusivity along the latitude and longitude sections in the BoB computed using Eq. (1). Eddy diffusivity obtained from this parameterization scheme shows large values (5 $\times$ 10${}^{-4}$ m${}^{2}$ s${}^{-1}$) in the north-central BoB where the internal tide focusing occurs. The values in the focal region are about 10 times larger compared to those in the open ocean and comparable to the values at the generation sites (Fig. 6b, c). This suggests that wave focusing significantly re-distribute the internal tide energy and their dissipation in the open ocean.

## Summary and Conclusions

Spatial variability of internal tides in the BoB is studied using satellite altimeter data. Large SSH signals with amplitudes of about 2.5 to 3.5 cm associated with M${}_{2}$ internal tides, observed near A & N Islands and adjacent to the continental slopes in the northern BoB suggest strong internal tide activity in these regions. One of the striking features of internal tide activity in this region is the presence of large amplitudes (about 3 cm) of SSH signals associated with M${}_{2}$ component in the north-central BoB, which is about 450 km away from the nearby generation sites. Simulations from a high-resolution numerical model show that the intensification of internal tides in the north-central BoB is due to the focusing of internal tides coming from multiple sources. These sources include the arc-shaped continental slopes in the northern BoB and the northern parts of the Andaman Islands (Preparis Channel). Relatively large oscillations of isopycnals (about 23 m for M${}_{2}$) and enhanced energy dissipation, which are even larger than those in the internal tide generation sites, are identified in the focal region.

Previous studies suggest that long-range propagating internal tides lose only a small portion of their energy in the open ocean^31,32^. Recently, Wang *et al*.^11^ showed that interaction of internal tides from multiple sources can cause inhomogeneity in the energy flux and dissipation in the open ocean. Our estimates based on numerical simulations suggest that focusing of internal tides from different sources to the north-central BoB re-distribute their energy in the open ocean and the convergence zones of internal tides may be hotspots of enhanced energy dissipation. Since the dissipation of internal tides being one of the primary sources for deep ocean mixing, such enhanced energy dissipation can result in increased vertical mixing. Therefore, identification of the focusing of internal tides and subsequent amplification in the open ocean regions will help us to interpret the *in-situ* observations on ocean mixing. Though, the present study is mainly based on the observations from satellite altimeters and numerical simulations, previous studies have shown that the spatial distribution of dissipation derived from model simulations were consistent with the observed variability of dissipation rate^33^. Direct observational evidence is  required to confirm this enhanced dissipation and associated mixing in the convergence zones in the north-central BoB.

## Data and Models

### Satellite altimeter data and extraction of tidal signal

Satellite altimeters can detect surface signatures of internal tides, which are of the order of few centimeters in the surface elevation^9,17^. The altimeter data of TOPEX/Poseidon (T/P) satellite mission is available from 1992 to 2006, Jason-1 (J1) from 2002 to 2008 and Jason-2 (J2) from 2008 to 2014. After the launch of J1 (J2) satellite, orbit of TP (J1) satellite was shifted to a secondary orbit (known as interleaved/tandem orbits (TPN + J1N)) and continued the data collection from 2002 to 2005 (2009 to 2012). All the satellites have a repeat cycle of 9.156 day and along-track resolution is 6.2 km^17,34^. Since the repeat cycles of satellites are much longer than semidiurnal and diurnal tidal periods (approximately 0.5 and 1 day), tidal signals were extracted using harmonic analysis from SSH by aliasing tidal periods to long periods (semidiurnal M${}_{2}$ lunar and S${}_{2}$ solar tide at 62.10 and 58.74 days). We use tidal constituents derived from time-combined altimetry records of T/P, J1 and J2 satellites, prepared by the Center for Topographic studies of the Ocean and Hydrosphere (CTOH/LEGOS), France (http://ctoh.legos.obs-mip.fr/ctoh). Amplitude and phase values for the tidal constituents are derived from Sea Surface Height (SSH) data that contain barotropic and internal tide signals which are phase locked to tidal frequencies (Fig. 1b). Since both barotropic and internal tides are confined to the same frequency, it is inseparable in the frequency domain. But the wavelengths of the barotropic (for example, ~9000 km for M${}_{2}$ tide if the depth of the ocean is 4000 m) and internal tides (typically less than 300 km for semidiurnal frequencies) are different and a spatial high-pass filter can be used to remove the signals of barotropic tides^9^. Theoretical wavelength of the first mode M${}_{2}$ internal tide in the BoB is about 100–130 km. Hence, we used a spatial high-pass filter with a 300 km cut off wavelength to remove barotropic tides which is much larger than theoretical wavelengths of first baroclinic mode semidiurnal internal tide in the BoB. Theoretical wavelengths of different baroclinic modes were determined using vertical profile of temperature and salinity (from WOA2009 climatology and model) by normal mode decomposition method^3^.

### Model simulations

Hourly snapshots from the simulations of a high-resolution (1/48${}^{\circ }$) ocean circulation model, configuration based on the Regional Ocean Modeling System (ROMS^35^) described in Jithin *et al*.^36^, for a period of one year (2013) are used in this study. The model domain extends from 77${}^{\circ }$E to 99${}^{\circ }$E and 4${}^{\circ }$N to 23${}^{\circ }$N, which covers the BoB and Andaman Sea (Fig. 1a). The initial and boundary conditions required for integrating the model are obtained from the simulations of a basin scale ROMS configuration for the Indian Ocean at $\sim$9 km resolution^36^. Barotropic tidal elevation and velocities in the model are forced at the open boundaries using 10 tidal constituents (M${}_{2}$, S${}_{2}$, N${}_{2}$, K${}_{2}$, K${}_{1}$, O${}_{1}$, P${}_{1}$, Q${}_{1}$, Mf and Mm) derived from the TPXO7.0 global tidal model^37^. Comparison of the model simulated barotropic and internal tides on the continental shelves and slopes in the western BoB with ADCP observations shows that the model has a very good skill in simulating coastal currents and internal tides in these regions^14,36,38^. Tidal currents and elevations from the model simulations are extracted by harmonic analysis using TUGOm Tidal ToolBox^39^. As noted earlier, satellite altimeter can detect signals which maintain a constant amplitude and phase for a long period of time (coherent internal tide) only. Therefore, spatially filtered SSH signals (high-pass filter with a 300 km cut off wavelength) obtained from the harmonic analysis of one-year sea level simulation are used for comparison with observed SSH signals from the satellite altimeter.

### Estimation of energy conversion, flux and dissipation

Barotropic tidal currents are defined as depth-averaged velocity fields and baroclinic currents are defined as the difference between total and depth-averaged currents. Both barotropic and baroclinic currents are subjected to harmonic analysis to extract the barotropic and baroclinic tidal currents and their amplitude and phase values. Barotropic tidal energy flux ${{\bf{F}}}_{{\bf{b}}}$ is estimated using the following expression,

2 $$\begin{array}{rcl}{F}_{bx} & = & \frac{1}{2}\rho gHU\zeta cos({\phi }_{\zeta }-{\phi }_{U})\\ {F}_{by} & = & \frac{1}{2}\rho gHV\zeta cos({\phi }_{\zeta }-{\phi }_{V})\end{array}$$

where $\zeta$ is the amplitude of surface elevation, $\rho$ is the reference density, $U$ (${\phi }_{U}$) and $V$(${\phi }_{V}$) are the amplitudes (phases) of northward and eastward components of tidal currents respectively^40^.

Following Kelly and Nash^41^, barotropic to baroclinic energy conversion rate ($C$) is computed using the relationship,

3 $$C=\left\langle \nabla H\cdot {{\bf{U}}}_{bt}\ {p}_{b}^{{\prime} }\right\rangle $$

where $dh$/$dx$ and $dh$/$dy$ are the bathymetric slopes in the east-west and north-south directions, ${U}_{bt}$ is the barotropic tidal current and ${p}_{b}^{{\prime} }$ is the baroclinic pressure perturbation at the bottom. Depth-integrated baroclinic energy flux (${\bf{F}}$) is estimated using the expression,

4 $${\bf{F}}=\underset{-H}{\overset{0}{\int }}\left\langle {\bf{u}}{\prime} (z)\ p{\prime} (z)\right\rangle dz$$

where ${\bf{u}}{\prime}$ and $p{\prime}$ are the baroclinic velocity fluctuation and baroclinic pressure anomaly respectively. Angle bracket (<>) indicates average over a tidal period^3,42^.

Depth-integrated energy density ($E$) is the sum of Horizontal Kinetic Energy ($HKE$) and Available Potential Energy ($APE$), given by

5 $$HKE=\frac{{\rho }_{0}}{2}\underset{-H}{\overset{0}{\int }}\left\langle {u{}^{{\prime} }}^{2}(z)+{v{}^{{\prime} }}^{2}(z)\right\rangle dz$$

6 $$APE=\frac{{\rho }_{0}}{2}\underset{-H}{\overset{0}{\int }}\left\langle {N}^{2}(z)\ {\eta }^{2}(z)\right\rangle dz$$

where ${\rho }_{0}$ is the vertically averaged water density, $u$′ and $v$′ are the baroclinic velocity fluctuations, $N$ is buoyancy frequency, and $\eta$ is the isopycnal displacement^3^.

Assuming that tendency and advection of internal tide energy are small. dissipation ($D$) of internal tides is estimated as

7 $$\nabla \cdot {\bf{F}}+D=C$$

here $C$ is the energy conversion and $\nabla \cdot {\bf{F}}$ is the divergence of depth-integrated energy flux^43,44^. It may be noted that $D$ could be somewhat overestimated as it includes the other budget terms (advection and tendency) and numerical dissipation. However, this method is widely used to estimate the baroclinic energy dissipation. Alford *et al*.^33^ noted that the spatial variability of observed dissipation is consistent with those estimated from the model simulation using Eq. (7). In addition, Mohanty *et al*.^13^ reported that tendency and advection terms are small compared to other terms along the AN Islands and Andaman Sea. Barotropic M${}_{2}$ tide loses about 65 GW of tidal energy in the entire model domain, in which about 28.5% (19 GW) of this energy is lost by internal tide dissipation and rest of the energy is dissipated by means of bottom friction. This suggests that the energy budget is closed inside the model domain.

## Supplementary information

Supplementary Information.

Supplementary Information 2.
