# Supplementary figures and images for: Intensification of tidally generated internal waves in the north-central Bay of Bengal
Source: Sci Rep. 2020 Apr 8;10:6059. doi: 10.1038/s41598-020-62679-4 (PMC7142159; doi:10.1038/s41598-020-62679-4)

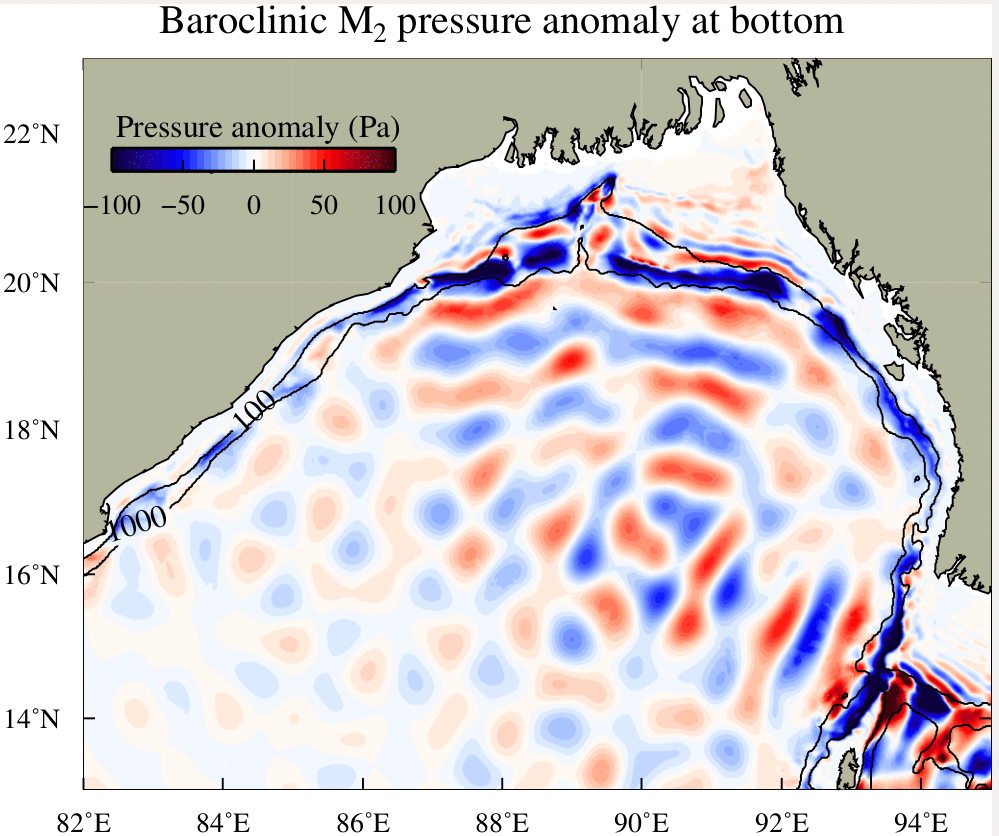

Supplement: Supplementary file 1 — Supplementary Information. [file 41598_2020_62679_MOESM1_ESM.gif]
