# Supplementary material for: Intensification of tidally generated internal waves in the north-central Bay of Bengal
Source: Sci Rep. 2020 Apr 8;10:6059. doi: 10.1038/s41598-020-62679-4 (PMC7142159; doi:10.1038/s41598-020-62679-4)
Supplement: Supplementary file 2 — Supplementary Information 2. [file 41598_2020_62679_MOESM2_ESM.pdf]

Supplementary information of

Intensification of tidally generated internal waves in the north-central Bay of Bengal

A.K. Jithin[1,3]., M.P. Subeesh[2]., P.A. Francis[1]., S.S.V.S. Ramakrishna[3]

[1] Indian National Centre for Ocean Information Services (INCOIS), Hyderabad, India, 500090
[2] National Centre for Polar and Ocean Research (NCPOR), Vasco da Gama, Goa 403804, India
[3] Department of Meteorology and Oceanography, Andhra University, Visakhapatnam-530003, Andhra Pradesh, India.
Corresponding Author: A.K Jithin., jithinoceanography@gmail.com

Supplementary 1

Animation of hourly bottom pressure anomaly patterns of M_2 internal in the northern BoB over M_2 tidal period (12.42 hours). Internal tides generated along the northern BoB radiate away from the sources and converge into the focal point centered at 89.35° E 16.4°N. [See the animation]

Supplementary 2

To understand the effect of stratification and background currents on the internal tide intensification in the north-central Bay of Bengal (BoB), we analysed monthly variation of internal tide energy flux, buoyancy frequency and surface currents in the region. Figure S2a & b show the depth-integrated M_2 energy flux in the northern BoB in the month of April and September respectively. Internal tides energy fluxes originating from the head of the bay are strong (about 3 kW m^{-1}) during September, whereas they are weak (about 1.5 kW m^{-1}) during April. This variation is mainly linked to changes in the internal tide generation due to the difference in background stratification in this region. Seasonal variation in the vertical stratification in the northern BoB are largely controlled by freshwater discharges from the adjacent continental rivers (Vinayachandran and Kurian 2007, Chaitanya et al.., 2014). Northern BoB receives maximum river discharge towards the end of southwest monsoon (September-November), which results in large near-surface stratification in this region (Vinayachandran and Kurian 2007, Papa et al., 2012). In the following months, stratification gradually decreases and near-surface stratification becomes weak during the period of March

to May. Buoyancy frequency in the head of the bay for the month of September and April are depicted in Fig. S2a to show the difference in vertical stratification. Even though there are differences in the strength of the internal tide beam originating from the major sources over time, focusing of internal tides and subsequent increase of internal tide energy in the north-central BoB exist throughout the year. However, the strength of internal tides in the focal region varies from month to month based on the strength of incoming beams from the adjacent sources.

One of the other mechanisms which can change the focusing of internal tide is the interaction of internal tides with background currents. Earlier studies showed that the background mesoscale eddy fields can alter the direction of propagation of internal tides (Huang et al., 2018, Dunphy et al., 2017). In light of this, we analysed the variation of internal tide radiation and associated intensification in the north-central BoB in the presence of monthly averaged mesoscale currents (Fig. S2). Monthly-averaged maps of currents computed from model simulations in the northern BoB show that the circulation in the northern BoB is dominated by mesoscale eddy fields. Spatial map of M_2 energy and flux show that though there are some slight deviations in the path of internal tides due to the presence of mesoscale circulations, there is no significant differences in the internal tide convergence and associated increase in the internal tide energy in the focal region. It may be noted that there are significant changes in the path of internal tides flux beam due to mesoscale current fields beyond the focal regions, especially in the fluxes that reach western BoB (Fig. S2b). The circulation (coastal currents) in the western BoB shows strong seasonal variation compared to those in the north-central BoB. In addition, the presence of mesoscale eddies also relatively large in the western BoB. This could be the reason for significant internal tide path alteration found in the western BoB. Jithin et al (2019) showed that a large part of internal tides observed in the continental margins of the western BoB come from Andaman-Nicobar Ridge. Hence, the path alteration of internal tide beam originating from the AN Ridge due to mesoscale fields could have a greater role in controlling the internal tide activity along the continental shelf and slope of the western BoB compared to north-central BoB.

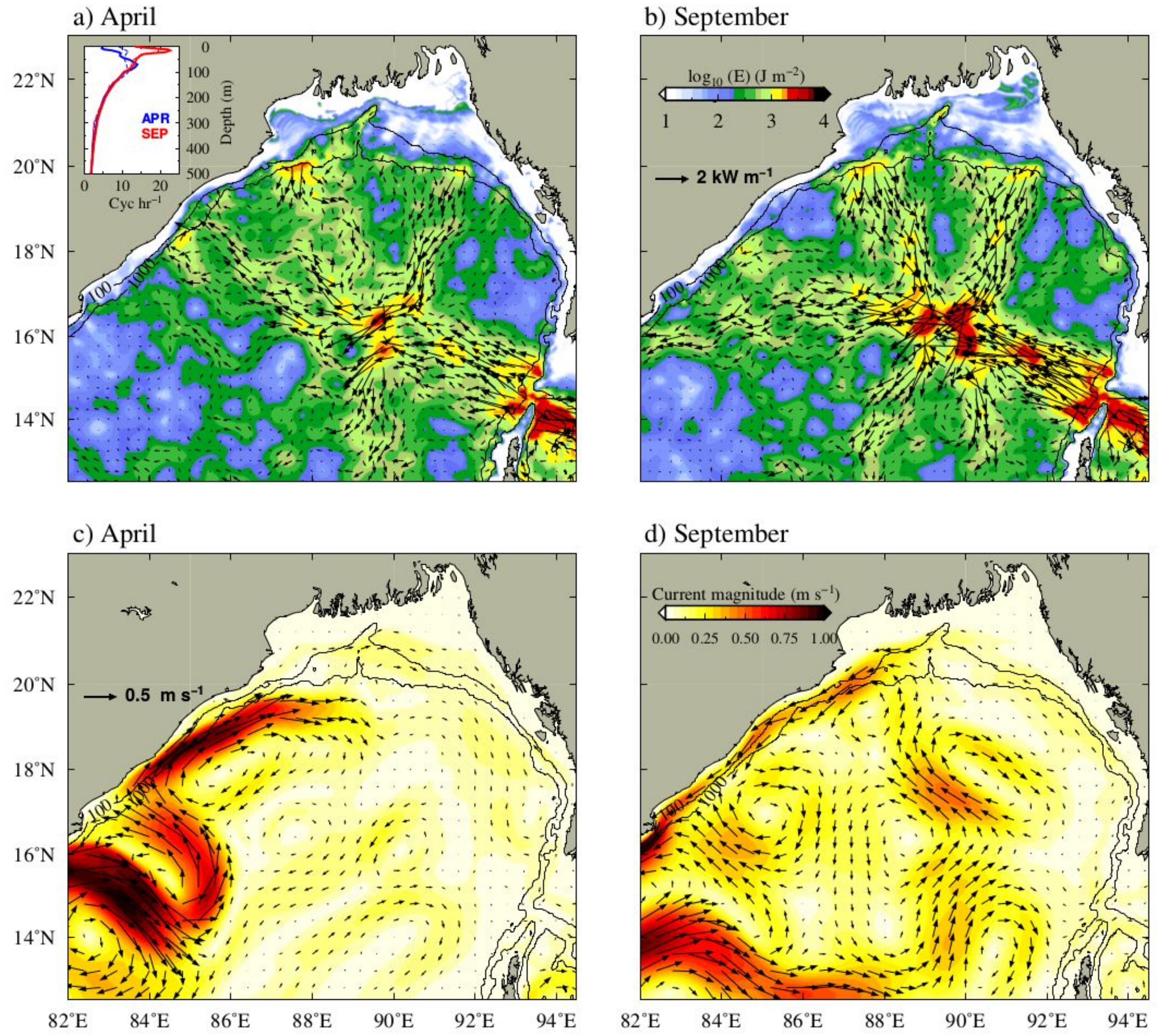

Fig. S2: Depth-integrated M_2 energy (logarithmic scale) and flux vectors in the northern BoB during (a) April and (b) September, 2013). Inset plot in (a) shows the buoyancy frequency for April (blue) and September (red) in the northern BoB. Model simulated near-surface current (0-50 m average) in the northern BoB during (c) April and (d) September. Bathymetric contours of 100 and 1000 m are also shown.

References for the supplementary information

Chaitanya, A. et al. Salinity measurements collected by fishermen reveal a “river in the sea” Flowing along the eastern coast of india. Bull. Am. Meteorol. Soc. 95, 1897–1908 (2014).

Dunphy, M., Ponte, A.L., Klein, P. and Le Gentil, S. Low-mode internal tide propagation in a

turbulent eddy field. *Journal of Physical Oceanography*, *47*(3), Pp.649-665 (2017).

Huang, X. et al. Role of mesoscale eddies in modulating the semidiurnal internal tide: Observation results in the northern South China Sea. J. Phys. Oceanogr. 48, 1749–1770 (2018).

Jithin, A., Francis, P., Unnikrishnan, A. & Ramakrishna, S. Modeling of internal tides in the western bay of bengal: Characteristics and energetics. J. Geophys. Res. Ocean. 124, 1 -27, DOI: 10.1029/2019JC015319 (2019).

Papa, F. et al. Ganga-brahmaputra river discharge from jason-2 radar altimetry: An update to The long-term satellite-derived estimates of continental freshwater forcing flux into the Bay of Bengal. J. Geophys. Res. Ocean. 117 (2012).

Vinayachandran, P.N. and Kurian, J. Hydrographic observations and model simulation of the Bay of Bengal freshwater plume. *Deep Sea Research Part I: Oceanographic Research Papers*, *54*(4), pp.471-486 (2007).
